# Genetic Analysis of IL-17 Gene Polymorphisms in Gout in a Male Chinese Han Population

**DOI:** 10.1371/journal.pone.0148082

**Published:** 2016-02-18

**Authors:** Zheng Zhou, Xinde Li, Hua Li, Mingzhen Guo, Shiguo Liu, Changgui Li

**Affiliations:** 1 Shandong Provincial Key Laboratory of Metabolic Disease, the Affiliated Hospital of Qingdao University, Qingdao, China; 2 Department of Rheumatology, the Affiliated Hospital of Qingdao University, Qingdao, China; 3 Prenatal Diagnosis Center, the Affiliated Hospital of Qingdao University, Qingdao, China; Kunming Institute of Zoology, Chinese Academy of Sciences, CHINA

## Abstract

Interleukin (IL)-17 is a proinflammatory cytokine mainly secreted by activated T helper 17 cells and involved in inflammatory immune responses. This study aimed to investigate the association between *IL-17* variants as well as serum IL-17 levels with gout in male Chinese Han individuals. A total of 1,101 male gout patients and 1,239 ethic-matched controls were enrolled. Genetic distributions of three variants (rs2275913 in *IL-17A*, rs763780 in *IL-17F*, and rs4819554 in *IL-17RA*) were detected by real-time polymerase chain reaction using the Taqman probe method. The plasma concentrations of IL-17A and IL-17F were measured in 228 gout patients and 198 controls that came from above samples by an enzyme-linked immunosorbent assay. No significant differences were observed in the genetic distribution of these polymorphisms between cases and controls (rs2275913: χ^2^ = 0.15, p = 0.928 by genotype, χ^2^ = 0.14, p = 0.711 by allele; rs763780: χ^2^ = 2.24, p = 0.326 by genotype, χ^2^ = 0.26, p = 0.609 by allele; rs4819554: χ^2^ = 1.79, p = 0.409 by genotype, χ^2^ = 1.46, p = 0.227 by allele). Levels of serum IL-17A and IL-17F were significantly decreased in gout patients (both p<0.001). However, no difference was observed in acute gout patients between different genotypic carriers of rs2275913 and rs763780 regarding serum IL-17A and IL-17F levels (p>0.05). Although the genetic variants in *IL-17* we studied in this research do not appear to be involved in the development of gout in male Chinese Han individuals, the IL-17 cytokine family may participate in gouty inflammation in an undefined way, which requires further validation.

## Introduction

Gout is an auto-inflammatory disease that occurs in the presence of elevated serum uric acid level and the precipitation of monosodium urate crystals (MSU) in joints and periarticular tissues. It is characterized by recurrent episodes of acute self-limiting arthritis. The incidence of gout has risen during recent years, commensurate with lifestyle and dietary changes, and affects 3.9% of the U.S. population, and 1.14% of those in the Shandong coastal cities of eastern China [1, 2].

Although it is the most common form of inflammatory arthritis, the pathogenesis of gout has not been fully clarified. Previous studies have indicated that gouty inflammation is mediated by the innate immune system, and marked by the recruitment of neutrophils into joints. Its pathogenesis is thought to be regulated by T cells through proinflammatory cytokines such as interleukin (IL)-1β, IL-6, IL-8, and tumor necrosis factor (TNF)-α [3, 4]. Gout is a polygenetic disease, and many studies of candidate genes have identified associations between inflammation-related genes such as *IL-1β*, *IL-6*, and *IL-8* and susceptibility to gout [5]. However, the results of these studies vary with different sample sizes and nationalities.

IL-17 is a proinflammatory cytokine mainly produced by T helper 17 (Th17) cells, which are a CD4^+^ T-cell subset [6]. The IL-17 cytokine family includes six subsets, IL-17A–F, which are encoded by *IL-17A–F*, respectively. IL-17A and IL-17F are responsible for mediating the immune inflammatory response in combination with their receptors. The IL-17 receptor (IL-17R) family comprises five receptor subunits, of which IL-17RA is the most important member. It is encoded by *IL-17RA*, located on chromosome 22q11.1, and is ubiquitously expressed in human T cells [7]. It forms a heterodimer with IL-17RC that binds to IL-17A and IL-17F [8]. As a potent inflammatory mediator, IL-17 recruits neutrophils and induces neutrophil-attracting chemokines to the site of inflammation, including IL-1, IL-6, IL-8, and TNF-α, thereby amplifying inflammatory responses [9]. Mills et al. previously demonstrated that cytokines such as IL-1β and IL-18 can act in synergy with IL-23 to promote IL-17A and IL-17F production by Th17 cells, which is thought to play a critical role in many auto-immune diseases [10].

Infiltration of the synovium by neutrophils is considered to be characteristic of gouty inflammation [7], and the development of acute inflammation in gout requires the participation of IL-17-related cytokines (IL-1, IL-6, IL-8, and TNF-α). We therefore hypothesized that these cytokines, particularly IL-17A and IL-17F and their corresponding receptors, might play important roles in the progress of gouty inflammation. The present study evaluated the association between *IL-17A* rs2275913, *IL-17F* rs763780, and *IL-17RA* rs4819554 single nucleotide polymorphisms (SNPs) as well as serum concentrations of IL-17A and IL-17F with gout susceptibility in a male Chinese Han population.

## Materials and Methods

### Subjects and clinical data

This study was conducted according to the ethical guidelines of the 1975 Declaration of Helsinki, and was approved by the Ethics Committee of the Affiliated Hospital, Qingdao University. All participants provided their written informed consent to participate in this study. We recruited 1,101 male gout patients from the Department of Gout, the Affiliated Hospital of Qingdao University, China between January 2009 and April 2015. Of these, 228 patients were categorized as acute gout (AG; n = 78) or with intercritical periods of gout (IG; n = 150). The diagnosis of gout was in accordance with American College of Rheumatology criteria [11], and the identification of different stages of disease was according to the European League Against Rheumatism guidelines for the diagnosis of gout in 2006 [12]. Patients with a history of other autoimmune diseases, nephropathy, cancer, or hematopathy were excluded. A total of 1,239 gout-free male controls with no other arthritis-related diseases were enrolled at the same time. All cases and controls were Chinese Han male individuals.

Blood samples were collected and immediately stored at −80°C for DNA extraction. Demographic and laboratory parameters, as well as medical history were carefully recorded by experienced endocrinologist physicians. These included the measurement of serum levels of uric acid, glucose, triglycerides (TG), total cholesterol (TC), creatinine, blood pressure, and body mass index (BMI).

### DNA extraction and genetic analysis

Genomic DNA from all participants was extracted from 200 μL peripheral blood samples using genomic DNA isolation kits (Qiagen, Hilden, Germany). Genetic distributions of the three variants (rs2275913, rs763780, and rs4819554) were detected by real-time PCR (CFX96^™^, Bio-Rad, Hercules, CA, USA) using the Taqman probe method. Primers were designed and synthesized by Applied Biosystems of Life Technologies (New York, USA). For rs2275913, forward and reverse primers were: 5′-TGCCCTTCCCATTTTCCTTCAGAAG-3′ and 5′-AGAGATTCTTCTATGACCTCATTGG-3′, respectively; for rs763780, these were: 5′-GTGGATATGCACCTCTTACTGCACA-3′ and 5′-GGTGGATGACAGGGGTGACGCAGGT-3′, respectively; and for rs4819554, these were: 5′-GGGAAGTAACGACTCTCTTAGGTGC-3′ and 5′-GCTGGGACACAGTCTCACAGACCAG-3′, respectively. PCR was carried out in 25-μL volume reactions containing: 12.5 μL PCR Master Mix (2×), 1.25 μL SNP Genotyping Assay (20×), 11.25 μL DNA sample, and DNase-free water to 25 μL. PCR conditions were: initial denaturization at 95°C for 3 min, followed by 40 cycles of denaturation at 95°C for 15 s and 60°C for 1 min. Genotypes were analyzed using Bio-Rad CFX manager 3.0 software.

### Determination of serum IL-17A and IL-17F levels

We collected the blood samples of 78 AG patients, 150 IG patients among all 1101 cases, and 200 controls among all 1239 control individuals, respectively. Serum was separated after centrifugation at 1000 × *g* for 15 min, then kept frozen at −80°C until assayed. Serum IL-17A and IL-17F levels were measured using a commercially available enzyme-linked immunosorbent assay (ELISA) kit according to the manufacturer’s instructions (Cusabio, Wuhan, China). Measurements were taken in duplicate to improve accuracy. The detection limits of IL-17A and IL-17F assays were 6.25–400 pg/mL and 12.5–800 pg/mL, respectively. Optical densities were measured with 450 nm wavelength using a hybrid multi-mode microplate reader (Synergy^™^ H1; BioTek, Winooski, VT, USA) with Gen5 Data Analysis Software. The standard curve was drawn by Curve Expert 1.4 software.

### Statistical analysis

Statistical analysis was implemented using the Statistical Package for Social Sciences version 22.0 (SPSS Inc., Chicago, IL, USA). The genetic distribution of the control group was tested for Hardy–Weinberg equilibrium (HWE) using a goodness-of-fit χ^2^ test. Differences in demographic and clinical indexes between two groups were compared using the Student’s *t*-test. Differences of polymorphisms between two groups were investigated using the χ^2^ test and P-values<0.017 were considered significant when Bonferroni’s correction was made. The strength of relationship in the allelic distribution between cases and controls was assessed by odds ratios and 95% confidence intervals. Power analysis was performed with the Power and Sample Size Calculations program, considering an alpha of 0.05, two-tailed. The analysis of variance was used to analyze differences in serum concentrations of IL-17A and IL-17F among three groups (AG, IG, and control groups) and the genotype–phenotype analysis among AG patients. We also made a bivariate correlation analysis of the serum IL-17A and IL-17F levels and the uric acid levels using Pearson correlation test. Statistical powers were two-sided at a significance level of 0.05, and differences were deemed to be significant at p<0.01.

## Results

### Demographic and clinical characteristics

Clinical characteristics of all subjects and p-values are summarized in Table 1. The mean ages of the case and control groups were 50.98 ± 13.92 and 59.72 ± 14.19 years, respectively. Gout patients were shown to have significantly higher diastolic pressure (85.01 ± 11.71 vs. 88.07 ± 12.46 mmHg, p<0.001), uric serum acid levels (313.87 ± 59.31 vs. 473.06 ± 122.44 μmol/L, p<0.001), serum TG levels (1.68 ± 4.44 vs. 2.36 ± 1.75 mmol/L, p<0.001), TC levels (5.33 ± 1.07 vs. 5.47 ± 1.35 mmol/L, p = 0.006), and creatinine levels (80.80 ± 17.52 vs. 88.80 ± 33.66 μmol/L, p<0.001) than controls. Other parameters such as BMI, systolic pressure, and blood glucose levels did not differ significantly between gout patients and controls (p = 0.967, p = 0.098, and p = 0.964, respectively; Table 1).

**Table 1:** Demographic and clinical characteristics of cases and controls.

characteristic	Gases (n = 1101)	Controls(n = 1239)	t	P
Age (years)	50.98 ± 13.92	59.72 ± 14.19	-14.59	<0.001
BMI (kg/m^2^)	27.00 ± 3.49	26.91 ± 70.00	0.04	0.967
Systolic pressure (mmHg)	135.47 ± 19.40	134.06 ± 19.61	1.66	0.098
Diastolic pressure (mmHg)	88.07 ± 12.46	85.01 ± 11.71	5.80	<0.001
Blood glucose (mmol/L)	6.16 ± 2.14	6.15 ± 7.02	0.05	0.964
Uric acid (μmol/L)	473.06 ± 122.44	313.87 ± 59.31	40.38	<0.001
Triglycerides (mmol/L)	2.36 ± 1.75	1.68 ± 4.44	0.49	<0.001
Total cholesterol (mmol/L)	5.47 ± 1.35	5.33± 1.07	2.78	0.006
Creatinine (μmol/L)	88.80 ± 33.66	80.80 ± 17.52	5.25	<0.001

BMI: Body Mass Index.

### Genetic analysis

The genetic distributions among controls of the three polymorphisms were in accordance with HWE (rs2275913: χ^2^ = 3.59, p = 0.058; rs763780: χ^2^ = 0.77, p = 0.382; and rs4819554: χ^2^ = 0.03, p = 0.867). Table 2 shows the genetic analysis of the whole study population, and demonstrates that there were no significant differences in genotypic or allelic frequencies for the three SNPs between cases and controls (rs2275913: χ^2^ = 0.15, p = 0.928 by genotype, χ^2^ = 0.14, p = 0.711 by allele; rs763780: χ^2^ = 2.24, p = 0.326 by genotype, χ^2^ = 0.26, p = 0.609 by allele; and rs4819554: χ^2^ = 1.79, p = 0.409 by genotype, χ^2^ = 1.46, p = 0.227 by allele). Subdivision of the samples into AG+GG/AA groups (for rs2275913 and rs4819554) or CC+CT/TT groups (for rs763780) also resulted in no differences between cases and controls for the three SNPs (all p>0.05). We also calculated the statistical power for the rs2275913, rs763780 and rs4819554 SNPs, power estimates of the allelic tests were 5.8%, 9.0%, and 13.5%, respectively.

**Table 2:** The genotypic and allelic frequencies of rs2275913, rs763780 and rs4819554 between cases and controls.

	Cases (n = 1101)	Controls (n = 1239)	χ^2^	P	OR(95%CI)
rs2275913					
Genotypes					
AA	229	250			
AG	510	576	0.15	0.928	
GG	362	413			
AG+GG	872	989			
AA	229	250	0.14	0.710	0.963(0.787–1.177)
Alleles					
A	968	1076			
G	1234	1402	0.14	0.711	1.022(0.910–1.148)
rs763780					
Genotypes					
CC	16	10			
CT	202	232	2.24	0.326	
TT	883	997			
CC+CT	218	242			
TT	883	997	0.03	0.871	1.017(0.829–1.248)
Alleles					
C	234	252			
T	1968	2226	0.26	0.609	1.050(0.870–1.268)
rs4819554					
Genotypes					
AA	357	434			
AG	553	601	1.79	0.409	
GG	191	204			
AG+GG	744	805			
AA	357	434	1.77	0.184	1.124(0.946–1.334)
Alleles					
A	1267	1624			
G	1469	1009	1.46	0.227	0.931(0.828–1.046)

### Association of serum IL-17A and IL-17F with gout

**Fig 1:**
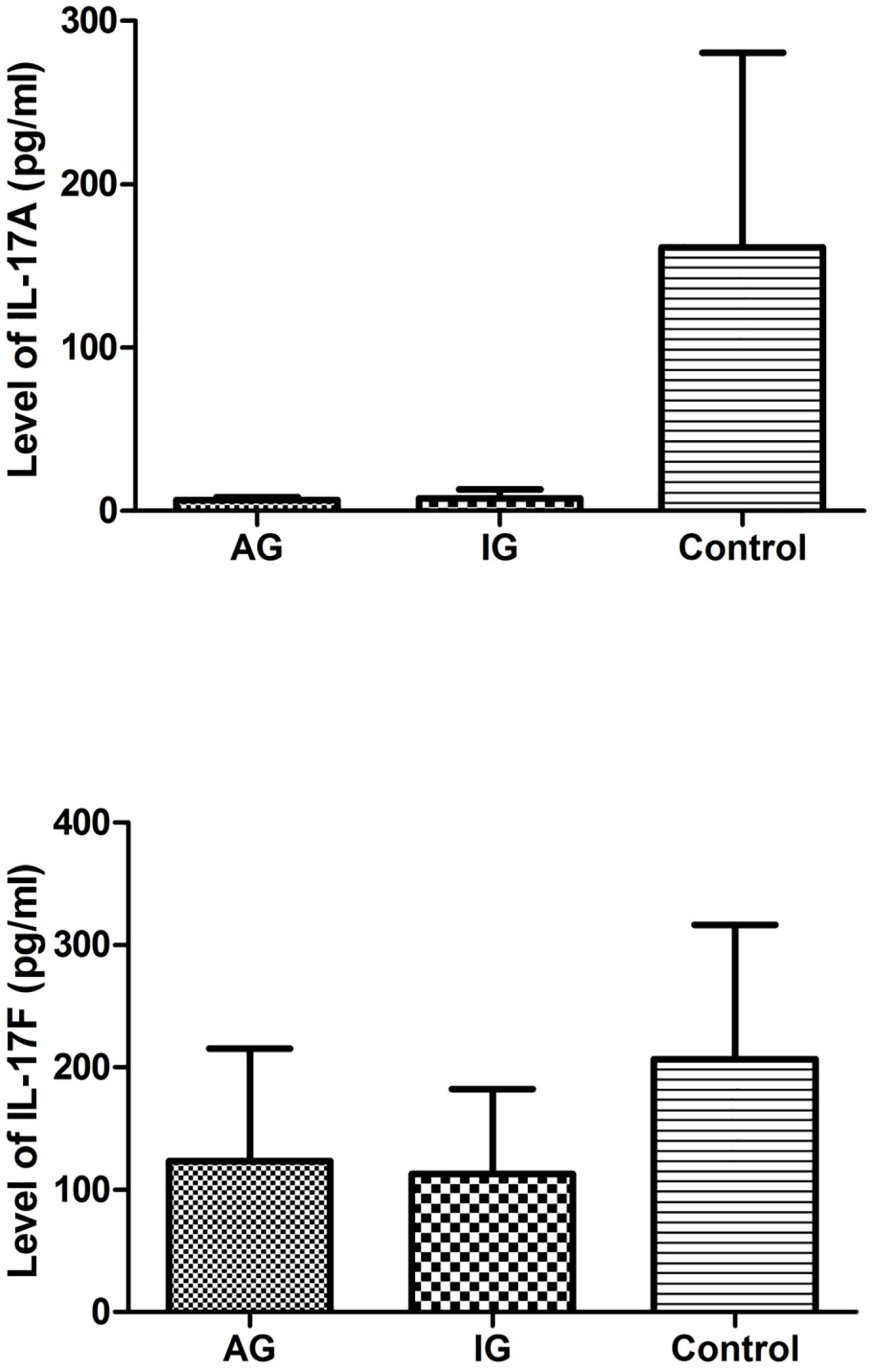
Levels of serum IL-17A and IL-17F in the AG, IG and control groups. Serum IL-17A and IL-17F levels of 78 AG patients, 150 IG patients and 198 control subjects were detected using Elisa kit. Expression of serum IL-17A and IL-17F were significantly decreased in the AG and IG group compared with controls (P<0.001; respectively), whereas no significant difference was observed between the AG and IG groups (both P>0.05). AG represents acute gout; IG represents intercritical periods of gout. The ANOVA and Bonferroni's test methods were used. The statistical significance was set at P<0.05.

As shown in Fig 1, mean IL-17A serum levels (± SD) were 6.74 ± 1.59, 7.92 ± 5.48, and 161.26 ± 119.27 pg/mL for AG patients, IG patients, and controls, respectively; for IL-17F, mean serum levels were 123.30 ± 92.32, 112.70 ± 69.87, and 206.80 ± 109.60 pg/mL, respectively. Thus, mean IL-17F levels were higher than those of IL-17A, but a similar tendency was observed for both in that they differed significantly among the three groups (χ^2^ = 173.38, p<0.001 and χ^2^ = 155.00, p<0.001, respectively). Serum IL-17A and IL-17F levels were significantly lower in AG and IG patients compared with controls (both p<0.001), whereas no significant difference was observed between AG and IG groups (both p>0.05).

**Fig 2:**
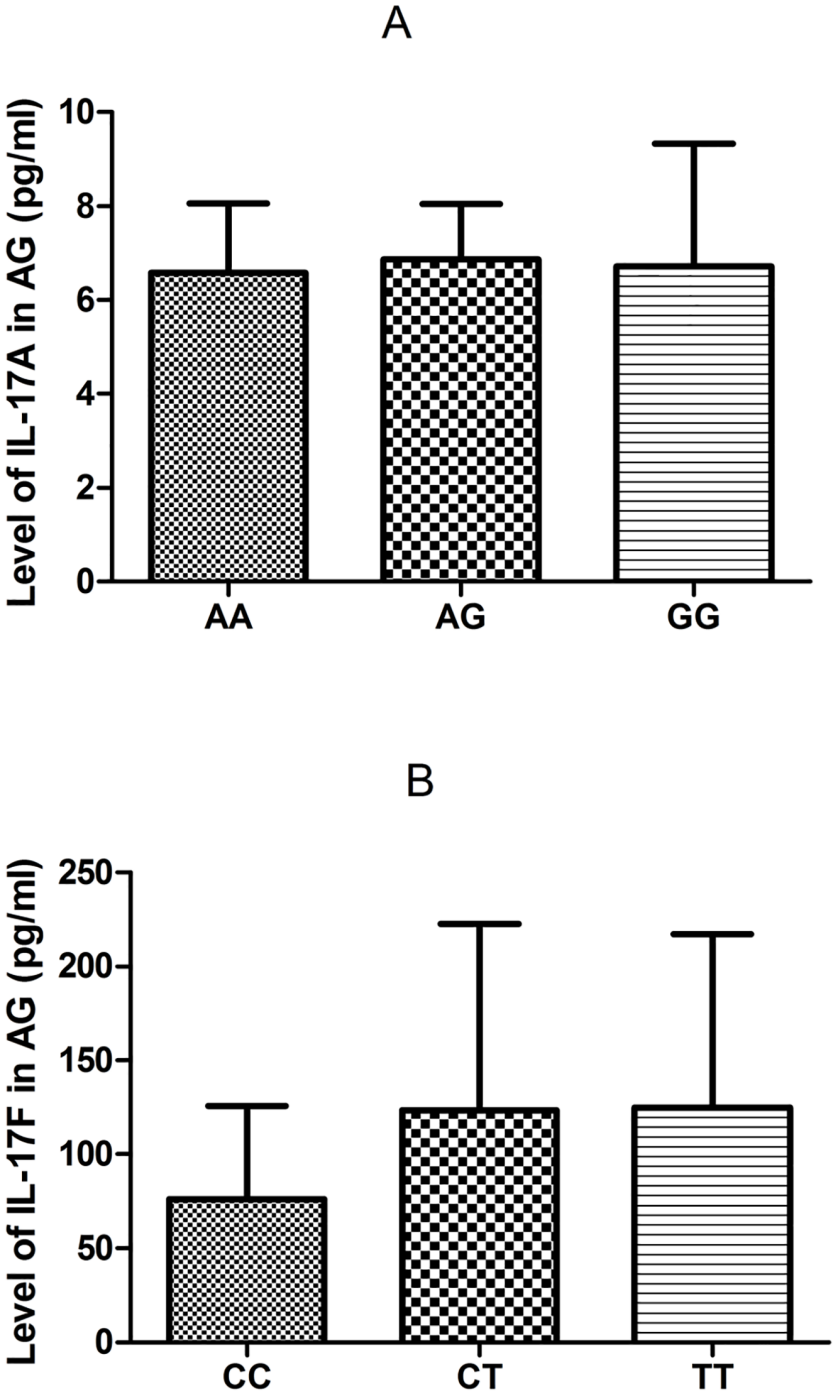
Association of the IL-17 polymorphisms with serum IL-17 levels in AG patients. The serum IL-17A and IL-17F level were measured in different genotype carriers from AG patients. A) The expression of IL-17A level was no difference between the 15 AA homozygotes, 24 AG heterozygotes carriers and 9 GG homozygotes carriers of IL-17A rs2275913 polymorphism. (P>0.05; respectively); B) The expression of IL-17F level was no difference between the 2 CC homozygotes, 14 CT heterozygotes carriers and 62 TT homozygotes carriers of IL-17F rs763780 polymorphism (P>0.05; respectively).

Fig 2 shows that no difference was observed in the expression of IL-17A or IL-17F among different genotype carriers (*IL-17A* rs2275913 AA, AG, and GG, and *IL-17F* rs763780 CC, CT, and TT) of AG patients (p>0.05). In addition, the correlation analysis of the serum IL-17A and IL-17F levels and the uric acid levels were tested, and the results showed no correlation was found (for IL-17A and the uric acid level, p = 0.443, for IL-17F and the uric acid level, p = 0.681).

## Discussion

Gout, characterized by the abrupt onset of severe joint pain, is an auto-inflammatory arthritis caused by abnormal serum uric acid level and the deposition of MSU crystals in synovial fluid and periarticular tissues. As an indicator of acute gouty arthritis, phagocytosis of MSU by macrophages can activate the NLRP3 inflammasome, which is considered to play a critical role in gouty arthritis. Caspase-1 is in turn activated by the NLRP3 inflammasome and lead to the conversion of pre-IL-1β and pre-IL-18 to mature IL-1β and IL-18. In the joint, the released IL-1β and IL-18 can bind to the IL-1 receptors in endothelial cells and resident macrophages, together with other pro-inflammatory cytokines and chemokines such as IL-6, IL-8, and TNF-α, finally lead to gouty inflammatory cascade [3, 13]. Several studies have focused on the relationship between gout and candidate inflammatory genes including *IL-1β*, *IL-6*, *IL-8*, *IL-18*, and *TNF-α* [13], however, the disease pathogenesis is still not fully understood.

The genes encoding IL-17A and IL-17F map to the same location on chromosome 6p12, and they share the highest degree of homology of the IL-17 cytokine family, being 50% identical [14]. They are expressed as disulfide-bonded homodimers [15], and can induce the expression of a series of inflammatory cytokines through their receptors IL-17RA and IL-17RC. They are therefore common to most inflammatory disorders, with previous pathological studies reporting the association of IL-17 with rheumatoid arthritis (RA), multiple sclerosis, psoriasis, inflammatory bowel disease (IBD), systemic lupus erythematosus, asthma, and Behçet’s disease [16]. Moreover, *IL-17* mRNA levels were shown to be elevated in asthmatic patients and to correlate with neutrophil aggregation [17, 18].

SNPs rs2275913 (−197G/A) located in the *IL-17A* promoter region and rs763780 (7488T/C) in exon 3 of *IL-17F* both influence *IL-17* expression [19], while the promoter SNP rs4819554 (−809A/G) regulates the expression and function of *IL-17RA* [20]. rs2275913, rs763780, and rs4819554 are all tag SNPs that have been associated with the pathogenesis of a variety of autoimmune and inflammatory diseases, including RA, IBD, asthma, and recurrent pregnancy loss [19–22]. Kawaguchi et al. previously reported that the expression and/or activity of *IL-17F* may be suppressed in *IL-17F* 7488C allele carriers and that the *IL-17F* His161Arg variant blocked the induction of IL-8 expression by wild-type IL-17F [22]. In the studies that based on Chinese Han population, Shu Q et al. [23] and Wang S et al. [24] found that rs763780 in IL-17F gene appears to be associated with susceptibility to both Vogt-Koyanagi-Harada (VKH) syndrome and multiple sclerosis. What’s more, Shen L et al. [25] suggested that IL-17A rs3819025 G/A and rs8193036 C/T variant alleles increase the risk of RA.

To our knowledge, this is the first study to investigate the association between *IL-17* variants and gout. Our results suggested that there is no association between the three SNPs and gout susceptibility. In our previous work, we identified associations between gout and SNPs in *IL-*17-related genes IL*-23R* rs7517847 [26] and *IL-8* −251T/A [27]. Because numerous variants are present within the *IL-17A*, *IL-17F* and *IL-17RA* genes, our selection of only three tag SNPs in the current study may explain our negative findings.

We divided the participants into three groups (AG, IG, and control groups). ELISA analysis showed that mean serum IL-17F levels were higher than those of serum IL-17A, and obviously decreased in AG and IG patients compared with controls, although no significant difference was observed between AG and IG groups. Mylona et al. previously reported that IL-17 levels were undetectable following stimulation of patient peripheral blood mononuclear cells with MSU [28], while Zhang et al. observed an elevated number of Th17 cells and serum IL-17 concentrations in RA patients compared with healthy individuals [29]. Kageyama et al. found that synovial fluid levels of IL-17 and IL-23 were significantly higher in RA patients compared with gout patients [30]. Our results indicated that IL-17 might be involved in the regulation of gouty inflammation, but the underlying regulatory mechanism appears to be ambiguous. Our findings are contrary to those of Zhang et al., perhaps indicating that the way in which IL-17 participates in gouty inflammation differs from its involvement in RA inflammation. We speculate that IL-17A and IL-17F together play a role in gout pathogenesis, which needs further investigation.

The present study has a number of limitations. First, all subjects were Chinese Han individuals from Shandong Province, so additional studies are required to determine the applicability of the results, given the important roles of both genetic and environmental factors in the development of gout. Second, the contributions of other important SNPs to gout susceptibility should also be investigated. Finally, the statistical power of this study was limited by its small sample size (for the rs2275913, rs763780 and rs4819554 SNPs, power estimates of the allelic test were 5.8%, 9.0%, and 13.5%, respectively). Although we found no association between the three SNPs and gout in the Chinese Han male population of the present study, further investigations are needed to determine the association between *IL-17* and *IL-17R* and gout using larger sample sizes, different populations, and other polymorphisms.
